# Loneliness and Changes in Cognitive Functioning Among Racially Diverse Older Adults in the United States

**DOI:** 10.1093/geroni/igab046.2251

**Published:** 2021-12-17

**Authors:** David Camacho, Kelly Pacheco, Charles Henderson, M Cary Reid, Elaine Wethington

**Affiliations:** 1 University of Maryland, Baltimore , Baltimore, Maryland, United States; 2 New York University, New York University, New York, United States; 3 Cornell University, Cornell University, New York, United States; 4 Weill Cornell Medicine, Weill Cornell Medicine, New York, United States; 5 Cornell University, Ithaca, New York, United States

## Abstract

Longitudinal studies examining the association of loneliness with cognitive decline rarely include older members of racial minorities. Guided by a Minority Stress Framework, we used Waves 2 and 3 from the National Social Life, Health, and Aging Project to assess whether loneliness (UCLA-3-items) at W2 predicts cognitive decline (Chicago Cognitive Functioning Measure) among US African-American, Latino, and white older adults (ages≥60). We included interactions of W2 loneliness with race in examining changes in cognitive functioning. Estimates were (N=1,950) adjusted for demographics, chronic disease, depression, and social connectedness. In all groups, loneliness was positively associated with greater global cognitive decline over the 5-year interval. However, analyses of different domains of cognitive functioning (e.g., executive functioning, memory) suggested that this association differs by cognitive domain and race. Future research on interventions to prevent cognitive decline should consider targeting loneliness, include diverse older adults, and examine global and specific domains of cognitive functioning.

